# Editorial: Understanding neural processing as an integrated intelligent system

**DOI:** 10.3389/fnsys.2026.1830216

**Published:** 2026-06-26

**Authors:** Paul J. Werbos, David S. Wack, Konstantinos Slavakis, Rajendra D. Badgaiyan

**Affiliations:** 1 Retired, Arlington, VA, United States; 2 Policy and Planning Committees of Millennium Project, Institute of Electrical and Electronics Engineers-USA, Washington, DC, United States; 3 Center for Large-Scale Complex Systems & Integrated Optimization Networks, The University of Memphis, Memphis, TN, United States; 4 Department of Radiology, Jacobs School of Medicine and Biomedical Sciences, University at Buffalo, Buffalo, NY, United States; 5 Department of Biomedical Engineering, University at Buffalo, Buffalo, NY, United States; 6 Department of Information and Communications Engineering, Institute of Science Tokyo, Tokyo, Japan; 7 Department of Psychiatry and Behavioral Sciences, Texas Tech University Health Sciences Center, Midland, TX, United States

**Keywords:** behavioral classification, brain connectivity, EEG, neural network modeling, post-concussion syndrome

Modeling brain circuits as intelligent systems addresses one of the most consequential gaps in contemporary science. Despite remarkable advances in neuroscience and artificial intelligence, a unified mathematical framework bridging biological data with machine learning and collective intelligence remains incomplete. The foundational systems neuroscience tradition—exemplified by Lashley (1929, 1950), Pribram (1991), and Freeman (1975, 2000)—studied the brain as a whole dynamical system possessing universal learning capability, unlike narrower approaches focused on isolated structures. The National Science Foundation's Cognitive Optimization and Prediction Networks initiative of 2007 (National Science Foundation, 2007) catalyzed cross-disciplinary research demanding balanced projects that advanced both brain understanding and artificial general intelligence, demonstrating that the deepest insights in both domains arise from the same intellectual soil. The articles collected in this Research Topic advance that integration across theory, tools, and clinical translation.

## 1 Novel mathematical models of brain circuit dynamics

Three articles in this Research Topic develop formal frameworks at different scales. Davis et al. extend Freeman's mesoscopic neurodynamics program (Freeman, 2000) with two complementary signal analysis methodologies—one based on the Hilbert transform, tracking how the brain participates in meaning creation through amplitude modulation patterns and phase transitions; one based on the Fourier transform, differentiating cognitive states through power spectral analysis. Both operate at the neuropil level, treating electromagnetic field dynamics across tens of thousands of neurons as the fundamental scale at which, in this tradition, meaningful brain activity becomes observable. This builds on Pribram (1991) insistence on accounting for field effects beyond axonal transmission, Buzsáki (2006) demonstrations that electromagnetic fields are crucial even for spike sorting, and Grossberg (2021) foundational computational models of neural mass dynamics. Kozma's continued development of Freeman's framework into the modeling of cognitive phase transitions in the cerebral cortex (Kozma and Freeman, 2016) extends this tradition.

Levine et al. bridge psychoanalytic theory with neural network dynamics, continuing a program with deep roots in systems neuroscience. Freud's *Project for a Scientific Psychology* (Freud, 1895/1950) described neural energy flowing through contact barriers of variable resistance—a scheme Pribram recognized as anticipating backpropagation. Levine et al. now advance a computational realization of this vision, modeling behavioral and emotional life as competing attractors in a system involving amygdala, prefrontal cortex, and hypothalamus. Their “wise ego” concept—balanced excitatory-inhibitory dynamics between orbitofrontal cortex and amygdala that suppress unhealthy emotional responses while enhancing healthy ones—gives psychoanalytic processes a rigorous computational formulation. Critically, the model treats therapeutic change as a nonlinear dynamical process: small shifts in the analyst–analysand interaction can move the system between attractors, and healthy adjustment requires maintaining multiple attractors rather than collapsing into a single rigid pattern.

Antonucci and Jay take a different clinical path with the Network Entrapment by Reflex Dysfunction model, proposing that persistent post-concussion symptoms arise not from diffuse cortical damage but from network entrapment: recursive maladaptive feedback loops across a five-node sensorimotor hierarchy involving reflex circuits, cerebellar calibration, basal ganglia gating, and cortical integration. When reflex-modulating structures are injured, distorted reafferent signals degrade gain modulation and overload thalamocortical systems, and the brain reorganizes into rigid but stable configurations—a maladaptive attractor state that deepens with each cycle unless specifically interrupted. This reframes treatment: restoring network dynamics through bottom-up sensorimotor intervention rather than managing symptoms at the cortical surface. Across all three contributions, the conclusion is consistent: reductionist single-structure explanations are insufficient. The brain is a dynamic hierarchical system whose behavior—healthy or pathological—emerges from network-level interactions.

## 2 Algorithmic tools bridging biology and machine learning

Three articles demonstrate the productive traffic now flowing across the brain–artificial intelligence bridge. Chowdhury et al. show that consumer-grade wearable biosensors combined with unsupervised machine learning can classify pharmacologically altered brain states—post-caffeine cortical arousal marked by alpha suppression and beta enhancement—without prior labeling, a proof-of-concept for real-time cognitive monitoring. Li et al. use active learning in their OpenLabCluster system to dramatically reduce the annotation burden in behavioral classification, validated across mice, zebrafish, *C. elegans*, and macaques—a species-agnostic tool providing critical infrastructure for comparative behavioral studies. Vidhusha et al. apply deep belief networks to electroencephalography-derived brain connectivity parameters—including power spectral density, Granger causality, and directed transfer function measures—achieving 89.7% accuracy classifying children with attention-deficit/hyperactivity disorder from typical children. Their framing of connectivity analysis not merely as diagnostic but as a measure of therapeutic effectiveness points toward a clinically important goal: quantifying how interventions change brain network dynamics in the developing brain.

What distinguishes these contributions is their grounding in systems neuroscience. The still-unexplained energy efficiency of biological neurons—a puzzle that motivated DARPA's SyNAPSE neuromorphic program (Merolla et al., 2014) and subsequent research by Kozma and Siegelmann among others (Saunders et al., 2018)—reminds us that algorithmic insight alone will not close the gap between neural and silicon computation. These are tools shaped by the conviction that oscillatory brain dynamics and behavioral patterns carry meaning that algorithms can help reveal.

## 3 What remains

These articles advance the systems neuroscience framework, but important territory remains uncharted. No contribution in this Research Topic addresses the temporal infrastructure of intelligence—the circuit-level timing precision on which cortical computation depends. Werbos and Davis (2016) probed those temporal patterns more deeply than any prior study, analyzing spike-sorted and burst-sorted data from Buzsáki's laboratory to deliver an empirical verdict among competing theories of cortical rhythms. Their circuit model—in which giant pyramid cells of neocortex receive clock pulses from the nonspecific thalamus at the apical dendrite (Werbos, 2009), a gating role for nonspecific thalamic systems documented in Schmitt and Worden (1974)—predicted the alpha-rhythm clock cycle and measured it to two-digit precision: 153.4 ms. The energy tradeoffs that distinguish levels of biological intelligence—computation vs. communication, the scaling laws that force architectural reorganization as brains grow—are implicit in several articles but explicitly modeled in none. Bitterman (1965) demonstration that intelligence differs qualitatively across species, not merely quantitatively, awaits the formal comparative framework that OpenLabCluster's cross-species infrastructure could eventually support. And the question of why mammalian neurons—even giant pyramidal cells—are so energy-efficient remains open. Extended Kalman Filter methods already yield orders-of-magnitude learning efficiency gains over backpropagation in recurrent networks (Ilin et al., 2008); Conrad's models of quantum molecular computing (Conrad, 1992) and the quantum field approach of Jibu and Yasue developed for Pribram's *Brain and Perception* (Pribram, 1991) suggest that a quantum extension of such methods could explain what classical architectures cannot—a possibility now approaching testability in quantum hardware. The neuron doctrine alone cannot account for what these articles collectively demonstrate: that the brain is a dynamic, hierarchical, field-generating, oscillatory, and meaning-creating system. That these contributions converge on attractor dynamics and network-level explanation across domains as different as psychoanalysis, post-concussion rehabilitation, and mesoscopic neurodynamics demonstrates the framework's reach. The gaps mark where the next advances must come.

## References

[B1] Bitterman M. E. (1965). The evolution of intelligence. Sci. Am. 212, 92–100. doi: 10.1038/scientificamerican0165-92 14252463

[B2] Buzsáki G. (2006). Rhythms of the Brain. Oxford: Oxford University Press.

[B3] Conrad M. (1992). Quantum molecular computing: the self-assembly model. Int. J. Quantum Chem. 44, 125–143. doi: 10.1002/qua.560440714

[B4] Freeman W. J. (1975). Mass Action in the Nervous System. New York, NY: Academic Press.

[B5] Freeman W. J. (2000). Neurodynamics: An Exploration in Mesoscopic Brain Dynamics. London: Springer. doi: 10.1007/978-1-4471-0371-4

[B6] Freud S. (1895/1950). “Project for a scientific psychology,” in The Standard Edition of the Complete Psychological Works of Sigmund Freud, Vol. 1 (London: Hogarth Press), 283–397.

[B7] Grossberg S. (2021). Conscious Mind, Resonant Brain: How Each Brain Makes a Mind. New York, NY: Oxford University Press. doi: 10.1093/oso/9780190070557.001.0001

[B8] Ilin R. Kozma R. Werbos P. J. (2008). Beyond feedforward models trained by backpropagation: a practical training tool for a more efficient universal approximator. IEEE Trans. Neural Netw. 19, 929–937. doi: 10.1109/TNN.2008.2000396 18541494

[B9] Kozma R. Freeman W. J. (2016). Cognitive Phase Transitions in the Cerebral Cortex: Enhancing the Neuron Doctrine by Modeling Neural Fields. Cham: Springer. doi: 10.1007/978-3-319-24406-8

[B10] Lashley K. S. (1929). Brain Mechanisms and Intelligence: A Quantitative Study of Injuries to the Brain. Chicago, IL: University of Chicago Press. doi: 10.1037/10017-000

[B11] Lashley K. S. (1950). “In search of the engram,” in Symposia of the Society for Experimental Biology, Vol. 4 (New York, NY: Cambridge University Press), 454–482.

[B12] Merolla P. A. Arthur J. V. Alvarez-Icaza R. Cassidy A. S. Sawada J. Akopyan F. . (2014). A million spiking-neuron integrated circuit with a scalable communication network and interface. Science 345, 668–673. doi: 10.1126/science.1254642 25104385

[B13] National Science Foundation (2007). Emerging Frontiers in Research and Innovation 2008 (EFRI-2008): Cognitive Optimization and Prediction (COPN) and Resilient and Sustainable Infrastructures (RESIN). Arlington, VA: National Science Foundation, Directorate for Engineering.

[B14] Pribram K. H. (1991). Brain and Perception: Holonomy and Structure in Figural Processing. Includes quantum field appendix by M. Jibu and K. Yasue. Hillsdale, MI: Lawrence Erlbaum Associates, Inc.

[B15] Saunders D. J. Siegelmann H. T. Kozma R. Ruszinkó M. (2018). “STDP learning of image patches with convolutional spiking neural networks,” in 2018 International Joint Conference on Neural Networks (IJCNN) (Rio de Janeiro: IEEE), 1–7. doi: 10.1109/IJCNN.2018.8489684

[B16] Schmitt F. O. Worden F. G. (eds.). (1974). The Neurosciences: Third Study Program. Cambridge, MA: MIT Press.

[B17] Werbos P. J. (2009). Intelligence in the brain: a theory of how it works and how to build it. Neural Netw. 22, 200–212. doi: 10.1016/j.neunet.2009.03.012 19386468

[B18] Werbos P. J. Davis J. J. J. (2016). Regular cycles of forward and backward signal propagation in prefrontal cortex and in consciousness. Front. Syst. Neurosci. 10:97. doi: 10.3389/fnsys.2016.00097 27965547 PMC5125075

